# Berberine modulates virulence gene expression in methicillin-resistant *Staphylococcus aureus* through the Agr system

**DOI:** 10.3389/fmicb.2026.1789015

**Published:** 2026-04-28

**Authors:** Fangfang Zhou, Qiang Xu, Wei Ren, Huijuan Cui, Xiaochun Sun, Lei Wang

**Affiliations:** 1 School of Medicine, Jiangsu University, Zhenjiang, Jiangsu, China; 2 Department of Laboratory, Shanghai Eighth People’s Hospital, Shanghai, China; 3 Department of Hepatology, Xinjiang Uygur Autonomous Region Hospital of Traditional Chinese Medicine, Urumqi, Xinjiang, China; 4 Department of Laboratory, Gansu Provincial Central Hospital, Lanzhou, Gansu, China; 5 Department of General Medicine, Shanghai Eighth People’s Hospital, Shanghai, China

**Keywords:** accessory gene regulator (Agr), berberine, biofilm, methicillin-resistant *Staphylococcus aureus*, virulence gene

## Abstract

Methicillin-resistant *Staphylococcus aureus* (MRSA) infection remains a major global health challenge, with its high pathogenicity largely attributed to a wide array of virulence factors. The accessory gene regulator (Agr) quorum-sensing system acts as a central hub for regulating virulence gene expression in MRSA, making it an attractive target for anti-virulence therapeutic strategies. This study investigated the inhibitory effect of the natural compound berberine (BBR) on MRSA virulence. Quantitative Real-time polymerase chain reaction (qRT-PCR) revealed that BBR significantly suppressed Agr system activity, leading to marked downregulation of key virulence genes, including *α*-Hemolysin (also known as α-toxin or Hla), Phenol-soluble modulin α (*psmα*), and Clumping factor A (*clfA*). Phenotypic assays confirmed that BBR treatment reduced hemolytic activity, biofilm formation, and host cell invasion. In a murine model of MRSA-induced pneumonia, BBR provided notable protective effects, as evidenced by reduced lung tissue damage, diminished inflammatory cell infiltration, and preservation of alveolar structure. Furthermore, BBR attenuated excessive production of pro-inflammatory cytokines and chemokines. Collectively, these findings demonstrate that BBR suppresses MRSA virulence by targeting the Agr quorum-sensing system. This highlights its potential as a novel anti-virulence agent or as a complementary approach to conventional antibiotic therapy, offering a promising strategy for managing MRSA infections.

## Introduction

*Staphylococcus aureus* is a highly prevalent commensal bacterium, colonizing 30–70% of the human population either transiently or persistently (Lin et al., 2024). In China, it is one of the most common bacterial pathogens and represents the leading cause of both community- and hospital-acquired infections, ranking first in isolation frequency among Gram-positive bacteria. Sputum specimens constitute the predominant source of isolates. At present, clinical practice predominantly identifies methicillin-resistant *Staphylococcus aureus* (MRSA) strains, which display broad-spectrum drug resistance (Cranmer et al., 2023). MRSA has emerged as a serious global public health concern. In 2022, the detection rate of MRSA in China was 28.9%, while Shanghai reported a rate of 43.8%—the second highest in the country and significantly above the national average. MRSA is capable of causing a wide range of infections, including suppurative lesions, pneumonia, pericarditis, sepsis, and other systemic diseases, largely through the secretion of diverse virulence factors (Krishna and Miller, 2012; Bergin et al., 2017; Smith et al., 2019). These include cell surface proteins such as Clumping factor A (ClfA), pore-forming toxins such as Panton-Valentine leukocidin (PVL), hemolysins, phenol-soluble modulins (PSMs), and serine proteases (SplB-SplF) (Chen et al., 2023). Collectively, these factors facilitate bacterial colonization, immune evasion, and subsequent disease progression. Among them, the surface protein ClfA, a fibrinogen-binding protein, plays a key role in plasma agglutination, mediating bacterial adhesion and promoting biofilm formation. ClfA also is an important virulence factor facilitating *S. aureus* bloodstream infections (Tkaczyk et al., 2016). Owing to these properties, ClfA is considered a promising target for controlling *S. aureus* infections (Lai et al., 2024).

The expression of virulence genes in *S. aureus* is regulated by the quorum-sensing (QS) system, a bacterial communication mechanism that detects population density (Paharik et al., 2016; González and Keshavan, 2006; Nakayama et al., 2013). The accessory gene regulator (Agr) system functions as the primary QS regulator in Gram-positive bacteria and is recognized as a key modulator of virulence factor expression, making it a promising target for therapeutic intervention. Related studies identified that genes regulated by the global virulence regulator Agr are important determinants of morbidity and mortality in the antibiotic treatment model of pneumonia. Several Agr-regulated genes have been implicated in pneumonia, including the staphylococcal *β*-barrel pore-forming toxins (Lacey et al., 2022). The Agr system is composed of two adjacent transcriptional units, RNAII and RNAIII (Thoendel et al., 2011). Upon stimulation by the autoinducing peptide (AIP), the histidine kinase AgrC activates the response regulator AgrA, which in turn promotes virulence factor expression through regulation of RNAIII (Queck et al., 2008). This intercellular communication plays a crucial role in bacterial survival, adaptation, and pathogenicity (Matsumoto et al., 2021; Whiteley et al., 2017).

Our previous study demonstrated that berberine (BBR) exhibits significant antibacterial activity against the MRSA standard strain USA300, exerting its bactericidal effects by altering cell membrane permeability and disrupting cell wall integrity (Zhou et al., 2024). Recent studies have revealed that berberine can inhibit MRSA biofilm formation by disrupting the aggregation of phenol-soluble modulins (PSMs) into functional amyloid fibrils. This interference subsequently enhances the bactericidal efficacy of co-administered antibiotics (Chu et al., 2016). Additionally, BBR can act as an anti-MRSA agent by modulating cell wall hydrolysis and regulating the expression of leukotoxins and other virulence factors (Gu et al., 2025). A growing number of small molecules have been described that down-regulate virulence gene expression in *S. aureus* without inhibiting growth, thereby exerting reduced selective pressure for resistance (Ganesan et al., 2023). Also, in a recent study, BCp12, a milk-derived AMP, is predicted to target the *S. aureus* cytoplasmic membrane, and also affects the QS system by down-regulating agrA, agrB, and agrC (Zhao et al., 2020; Li et al., 2022) and plant-derived compounds such as carvacrol and cinnamaldehyde, inhibited biofilm formation and virulence while enhancing the activity of *β*-lactam antibiotics against MRSA by targeting SarA (Li et al., 2026). Among these, CY-158-11, a novel small-molecule compound containing N-maleimide and diphenyldiselenide, effectively suppresses the expression of MRSA virulence genes, including *sarA*, *agrA*, *clfA*, *clfB*, and *psmβ* (Shen et al., 2023)*. A*lso CY-158-11 exhibiting antibacterial activity against *S. aureus in vitro* and *in vivo* at relatively low concentrations. Furthermore, the investigation of its mode of action revealed that CY-158-11 can selectively perturb the cytoplasmic membrane of bacteria without harming mammalian cells or mouse organs (Shen et al., 2024). These findings suggest that exploring novel agents capable of attenuating virulence factor expression may provide a more promising strategy for treating *S. aureus* infections. In this study, we investigated the impact of BBR on MRSA virulence genes and evaluated its inhibitory effects through suppression of virulence gene expression.

## Materials and methods

### Bacterial strains, cells, and growth conditions

The bacterial strain used in this study was USA300, kindly provided by the LAN Lefu Research Group, School of Pharmaceutical Science and Technology, Hangzhou Institute for Advanced Study, University of Chinese Academy of Sciences. *S. aureus* strains were cultured in tryptic soy broth (TSB; Difco) at 37 °C with shaking at 250 rpm or maintained on tryptic soy agar (TSA; Difco). L929 mouse fibroblast cells were cultured in RPMI-1640 medium supplemented with 10% fetal bovine serum (FBS; Sigma-Aldrich, St. Louis, MO, USA) at 37 °C in a humidified incubator containing 5% CO_2_.

### BBR preparation

BBR was purchased from Beijing WoKe Biological Technology Co., Ltd. (batch number: XW20868312) and dissolved in dimethyl sulfoxide (DMSO). The prepared BBR solution was used for both *in vitro* assays and *in vivo* experiments in mice.

### Spread plate assay

The antibacterial activity of BBR against USA300 was evaluated using the spread plate method. A bacterial suspension of USA300 was incubated with varying concentrations of BBR in LB medium at 37 °C with shaking (220 rpm) for 24 h. Subsequently, 10 μL of each culture was spread onto pre-prepared agar plates. After drying, the plates were incubated statically at 37 °C for 24 h. Following incubation, plates were imaged, and colony numbers were quantified using ImageJ software. Agar plates inoculated with the original bacterial suspension without BBR served as the control (Herigstad et al., 2001).

### Preparation of BBR antimicrobial susceptibility test discs

Aqueous extracts of BBR were prepared at concentrations of 128, 64, 32, 16, and 8 μg/mL. For each sterile blank antimicrobial susceptibility disc (Beckman Bio, China), 15 μL of the corresponding BBR solution was applied and allowed to absorb completely. Discs were then dried at 37 °C for 18 h. This procedure was repeated by adding an additional 15 μL of the same solution to the same disc, followed by drying under identical conditions. The fully dried discs were sealed in sterile glass bottles and stored at 4 °C until use.

### Disc diffusion method for measuring inhibition zone diameter

Pure *S. aureus* colonies cultured for 18–24 h were selected for testing. A bacterial suspension was adjusted to a turbidity equivalent to a 0.5 McFarland standard. Using a sterile cotton swab, the suspension was evenly spread over the surface of Mueller-Hinton (MH) agar plates. BBR antimicrobial susceptibility test discs were then placed on the inoculated agar surface. Plates were incubated at 35 °C for 18 h. The diameters of the inhibition zones were measured using a vernier caliper, and antibacterial activity was evaluated based on zone size.

### RNA extraction and qRT-PCR

Bacterial cultures were diluted to an initial OD₆₀₀ ≈ 0.1, treated with different concentrations of BBR, and incubated at 37 °C for 3 h. DMSO-treated cultures served as controls. RNA was extracted using the RNeasy Mini Kit (Qiagen), and cDNA was synthesized using the M-MuLV First Strand cDNA Synthesis Kit (Sangon Biotech). The reverse mRNA levels reaction conditions were: 42 °C for 30–60 min (cDNA synthesis) and 70 °C for 10 min (reaction termination). qRT-PCR was performed using 2 × SG Fast qPCR Master Mix (Low Rox; Sangon Biotech) on a QuantStudio 5 real-time PCR system (Applied Biosystems, Thermo Fisher Scientific). The thermal cycling program was as follows: 95 °C for 3 min (pre-denaturation), followed by 40 cycles of 95 °C for 3 s (denaturation) and 60 °C for 30 s (annealing/extension). Primer sequences are provided in Table 1. For controls, untreated samples served as the positive control for target genes; 16S rRNA was used as an internal reference gene to ensure stable normalization; and nuclease-free water was used as the negative control to exclude contamination. Gene expression changes were calculated using the 2^−ΔΔCt^ method. All experiments were performed with three independent biological replicates, and each gene was tested in triplicate (technical replicates) (Lu et al., 2023).

**Table 1:** Primer pairs used in qRT-PCR.

Gene	Primer sequence (5′-3′)
*clfA*-RT-F	CCAGAAAACTTTGAGGATGTCACTA
*clfA*-RT-R	TGCTATTCGGATCAATATGACCA
*RNAIII*-RT-F	AATACATAGCACTGAGTCCAAGG
*RNAIII*-RT-R	TGGATTATCGACACAGTGAACA
*SplB*-RT-F	TATGAGTCA ACTGGCCCTGTGATG
*SplB*-RT-R	CCAGAGTTTCCGCTTTCAGTATGC
*splC*-RT-F	ACTGCCCATCCAAACGGTGAC
*splC*-RT-R	CCACGT TCGACTGCTTGTTCTTC
*splD*-RT-F	CTGGTGTCGGCACAACAGTGG
*splD*-RT-R	CCAGCGCCCATCCATGTAACAC
*splE*-RT-F	TTTCGTAGAACCAGGCAACTCAGG
*splE*-RT-R	CGTTTCCACCAAAGTGAACACCTAC
16S rRNA-RT-F	CTGTGCACATCTTGACGGTA
16S rRNA-RT-R	TCAGCGTCAGTTACAGACCA
*splF*-RT-F	GGATGCAATTATTCAGCCTGGTAGC
*splF*-RT-R	TCCTCTTGTGCTTTCACCTGATGG
*sspA*-RT-F	ATGAAAGGTAAATTTTTAAAAGTTAGTTCTTTATTCT
*sspA*-RT-R	ATCTTCAATATTTTGTTTTAAGAAGTTGCGTACA
*sspB*-RT-F	GTAAATCTAGAGTATTCAATATTATCAGCATCATAATGG
*sspB*-RT-R	AACCTATCATTGAACCATACCAGTTATAATCA
*sarR*-RT-F	TGTTCAGAGTTCAAGCCTTACT
*sarR*-RT-R	ACGATTACTGTTCTTTCGTCGT
*Agr*-RT-F	ACAACCACAAGTTGTTAAAGCAG
*Agr*-RT-F	GTTGTTTGCTTCAGTGATTCGTT

### RNA-sequencing

Extracted RNA samples were sent to Shanghai Sangon BioEngineering Co., Ltd. for transcriptome library construction and sequencing using the Illumina HiSeq platform. Differentially expressed genes (DEGs) were identified using both DESeq2 and PossionDis algorithms. DEGs were screened with DESeq2 based on a fold-change ≥ 2 and an adjusted *p* value ≤ 0.05. Functional classification and enrichment analyses were performed using the clusterProfiler package.

### Molecular docking

The structure file of BBR (CAS: 2086-83-1) was obtained from the PubChem database. The structure was optimized into a 3D conformation using Chem3D v20.0 software with MM2 force field minimization and then exported in PDB format. Gene names corresponding to the core protein targets were retrieved from the UniProt database, and the predicted 3D structures of these proteins were downloaded in PDB format from AlphaFold. The BBR ligand and target proteins were processed in AutoDock Tools v1.5.7 for hydrogenation and charge calculation, followed by conversion into PDBQT format. Binding pockets were predicted using POCASA v1.1, and docking grids were set with dimensions of 40 Å × 40 Å × 40 Å. Molecular docking was performed with AutoDock Vina v1.2.5, and the best binding energy conformations were selected for further analysis. Binding energies were visualized as a heat map. Protein-ligand interactions were analyzed with PyMOL v2.5.4, focusing on hydrogen bonds formed with amino acid residues. Interaction diagrams were generated with proteins displayed in cartoon representation, ligands and interacting residues in stick representation, and hydrogen bonds highlighted with yellow dashed lines (Yuan et al., 2023). The names of the target proteins and their UniProt IDs are provided in Table 2.

**Table 2:** The names of the target proteins and the UniProt database IDs.

Protein name	UniProt ID	Center x	Center y	Center z
clfA	Q2G015	−2.2	4.3	2
sarR	Q9F0R1	−10.8	−4.1	4.6
splB	Q2FXC3	2.8	3.9	−9.8
splC	Q2FXC4	−10.8	16.9	−1.4
splD	Q2FXC5	13	−18.7	−33.5
splE	Q2FXC7	1.8	−5.7	−18.1
splF	Q2FXC8	13.4	−9.1	0.9
sspA	Q2FZL2	3.4	−1.7	−21.6
sspB	Q2FZL3	−5.2	−0.1	−18.9

### Biofilm assay

BBR was dissolved in DMSO at a stock concentration of 20 mg/mL. USA300 was inoculated into 5 mL of TSB and cultured at 37 °C with shaking at 220 rpm. BBR working solutions of different concentrations were prepared in TSB supplemented with 1% glucose and added to bacterial cultures in 96-well plates at a ratio of 1:100, with three replicate wells for each concentration. The same volume of DMSO was included as a solvent control. After 24 h of incubation, supernatants were discarded and wells were washed three times with phosphate-buffered saline (PBS). Biofilms were fixed with 99% methanol for 15 min, followed by removal of the fixative. Biofilms were then stained with 2% crystal violet for 10 min and rinsed under running water until the wash was clear. After air drying, 70% glacial acetic acid was added to solubilize the stain, and absorbance at 590 nm was measured. Biofilm structures were further examined using a confocal laser scanning microscope (Chung et al., 2022). TSB served as the blank control. All experiments were performed in triplicate.

### Congo red agar (CRA) method

Production of amyloid fibrils by *S. aureus* was assessed using the CRA method described by Freeman et al. The medium consisted of TSB (37 g/L), sucrose (50 g/L), and Congo red (0.8 g/L; AMERCO, Solon, OH, USA). Congo red was prepared as a concentrated aqueous solution, sterilized separately by autoclaving at 121 °C for 15 min, and added to the medium once cooled to 55 °C. Plates were inoculated and incubated aerobically at 37 °C for 24 h. Colonies producing amyloids appeared red with a dry, crystalline consistency, while non-amyloid-producing colonies remained pink, often with central darkening (Xiong et al., 2022).

### MTT assay

The cytotoxicity of BBR was assessed in L929 cells using the MTT assay. L929 cells (1 × 10^5^ cells/mL) were seeded into 96-well plates and cultured for 24 h. Cells were then treated with BBR at concentrations of 0, 16, 32, 64, 128, and 256 μg/mL for 24 h. After treatment, MTT solution was added to each well and incubated for 4 h. The resulting formazan crystals were dissolved in DMSO (Sigma-Aldrich), and absorbance was measured at 490 nm using a microplate spectrophotometer (Epoch, BioTek, Winooski, VT, USA). Each experiment was performed in triplicate (Mosmann, 1983).

### MRSA-induced mouse pneumonia model and BBR treatment

The MRSA pneumonia model was established based on previously reported methods with minor modifications. Female ICR (CD1) mice (20–22 g) were intranasally inoculated with USA300. The bacterial inoculum was prepared at 1 × 10^9^ CFU/mL, and 50 μL of suspension was instilled intranasally once daily for three consecutive days to induce pneumonia. The mice were randomly divided into five groups, with five mice in each group. Survival was monitored twice within the first 24 h post-infection and once daily thereafter until day 8. Mice administered normal saline served as negative controls, while untreated infected mice were designated as the model group. The treatment group received BBR at a dose of 0.04 mg/g. Upon death or completion of the study, organs (heart, liver, spleen, lungs, and kidneys) were collected, weighed, and homogenized for CFU enumeration. Peritoneal lavage was performed by injecting 3 mL of saline into the abdominal cavity, followed by gentle massage; the lavage fluid was then collected with a syringe. Female ICR (CD1) mice were purchased from Zhejiang Vitariver Laboratory Animal Technology Co., Ltd. All animals were housed under specific pathogen-free conditions at the Shanghai Institute of Immunology and Infection, with ad libitum access to food and water. Environmental conditions included a 12-h light/dark cycle, a temperature of 20–26 °C, and a relative humidity of 40–60% (Li et al., 2023). All animal protocols were reviewed and approved by the Animal Care and Use Committee of the Xinjiang Uygur Autonomous Region Institute of Traditional Chinese Medicine (Approval No. IACUC-20250426-41) and conducted in accordance with institutional and national ethical guidelines.

### Enzyme-linked immunosorbent assay (ELISA) of mouse serum

To determine cytokine concentrations, whole blood was collected from mice and allowed to clot at room temperature. Samples were centrifuged at 1000 × *g* for 20 min at 4 °C, and the resulting serum was collected for analysis. Levels of IL-6, IL-1β, and TNF-α in serum were quantified using commercial ELISA kits (Shanghai Sangon Biotech Co., Ltd.).

### Statistical analysis

All statistical analyses were performed using GraphPad Prism 8.0 software. Data were analyzed using Student’s *t*-test or one-way analysis of variance (ANOVA), as appropriate. A *p*-value < 0.05 was considered statistically significant (*p* < 0.05, *p < 0.01).

## Results

### BBR inhibits the growth of USA300

**Figure 1:**
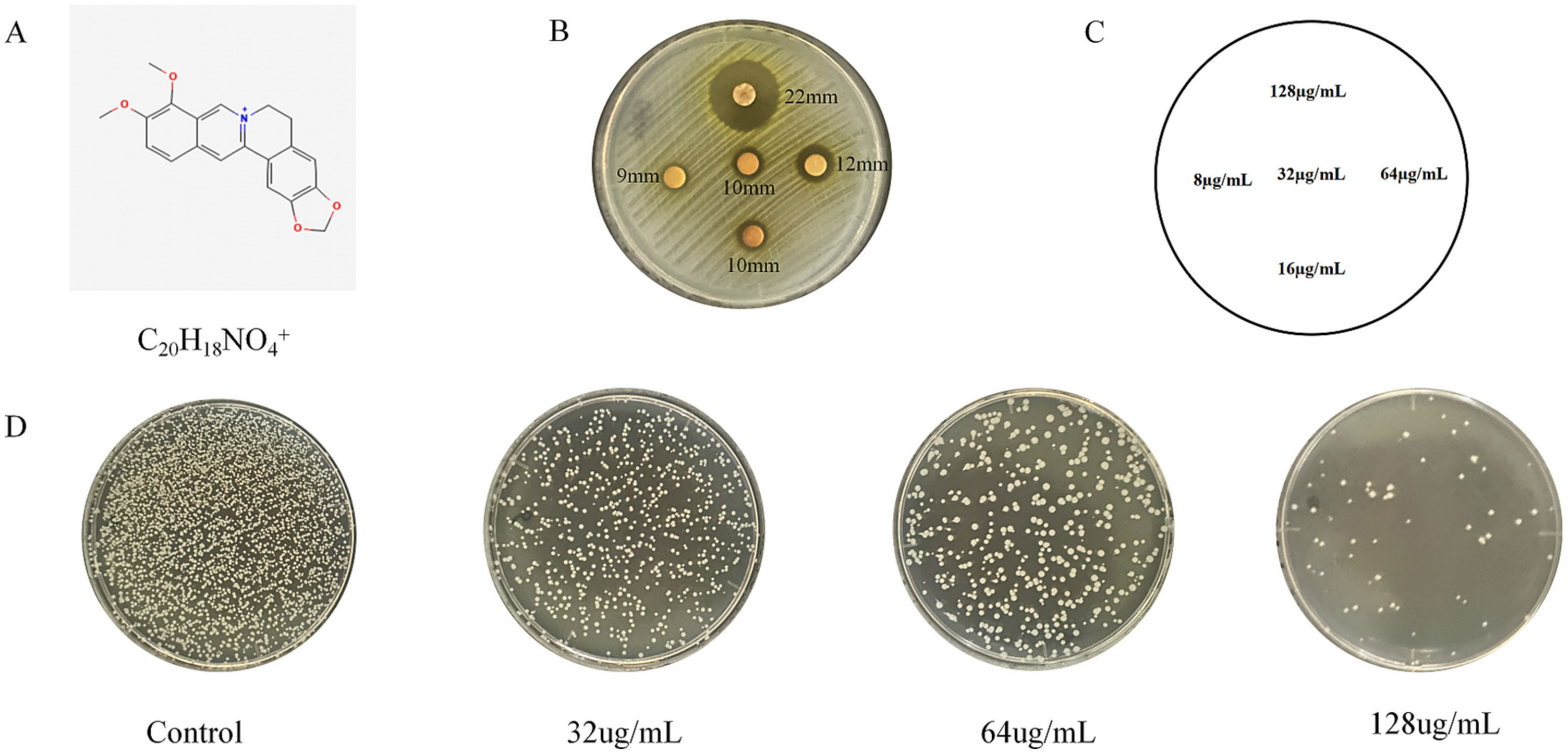
BBR inhibits the growth of USA300. **(A)** The chemical structural formula of BBR; **(B)** BBR antimicrobial susceptibility test discs; **(C)** describes the images for **(B)**; **(D)** spread plate.

Berberine, a quaternary ammonium alkaloid derived from the traditional Chinese medicinal plant Coptis chinensis, serves as the primary active component responsible for its antibacterial properties. The molecular formula of berberine is C_20_H_18_NO4^+^, as shown in Figure 1A. The minimum inhibitory concentration (MIC) of BBR against the USA300 strain was determined to be 128 μg/mL (Gu et al., 2025). As shown in Figure 1B, BBR exhibited no bactericidal activity at concentrations between 8 and 16 μg/mL, but demonstrated a clear bacteriostatic effect on MRSA at 64 and 128 μg/mL in agar plate assays. The mean diameters of the inhibition zones at 64 and 128 μg/mL BBR were 12 mm and 22 mm, respectively. Spread plate analysis further revealed that 16 μg/mL BBR significantly inhibited USA300 growth, while 128 μg/mL BBR nearly completely suppressed bacterial proliferation, as shown in Figures 1C,D.

### BBR affects the mRNA levels of Agr in USA300

**Figure 2:**
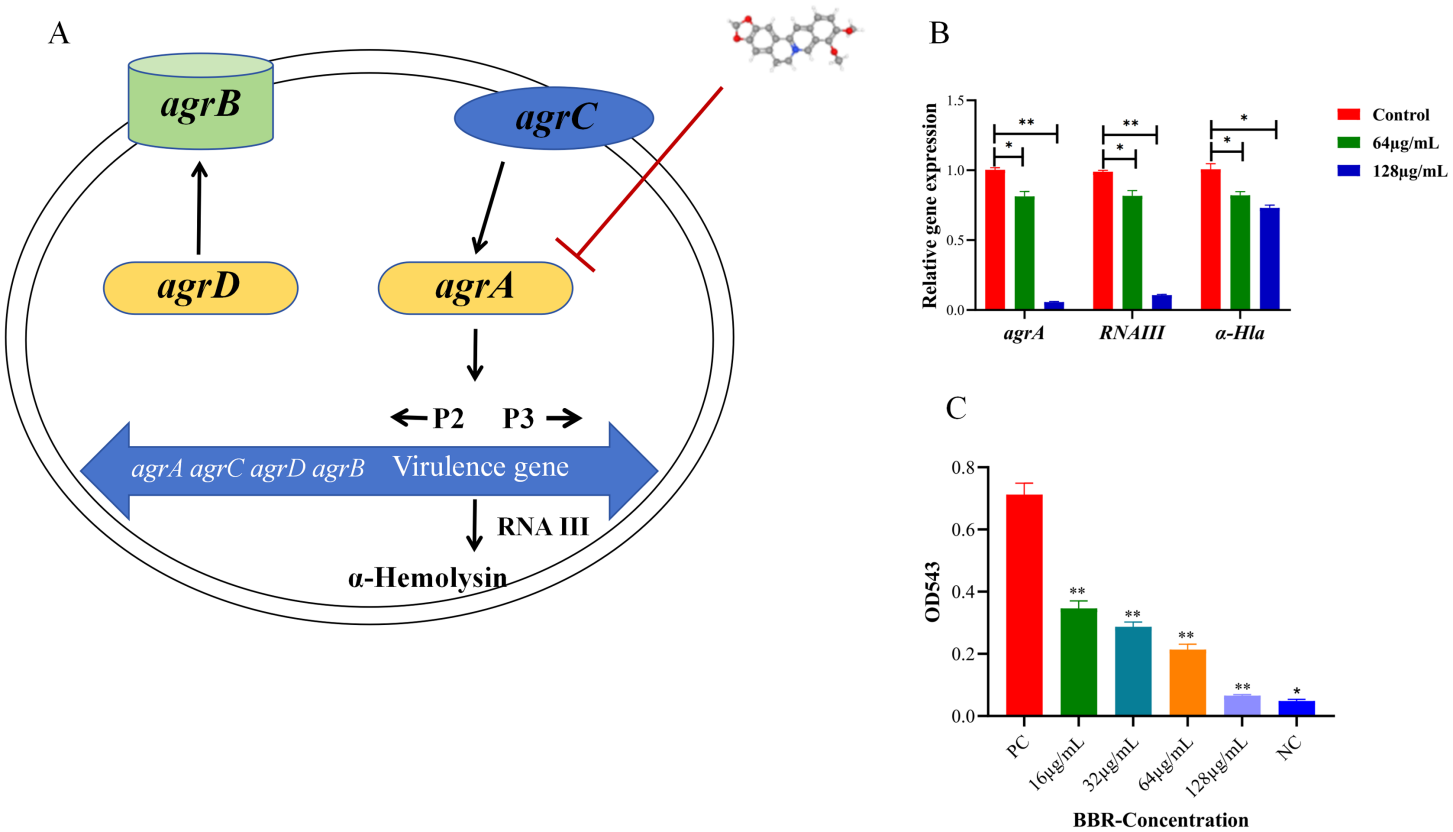
The influence of BBR on Agr expression. **(A)** Schematic representation of the mechanism of BBR on the Agr system; **(B)** qRT-PCR was used to verify the effect of BBR on Agr; **(C)** Various concentrations of BBR were used to treat mouse red blood cells, and the hemolysis was calculated. NC, Negative control; PC, positive control. **p < 0.01, *p < 0.05.

**Figure 3:**
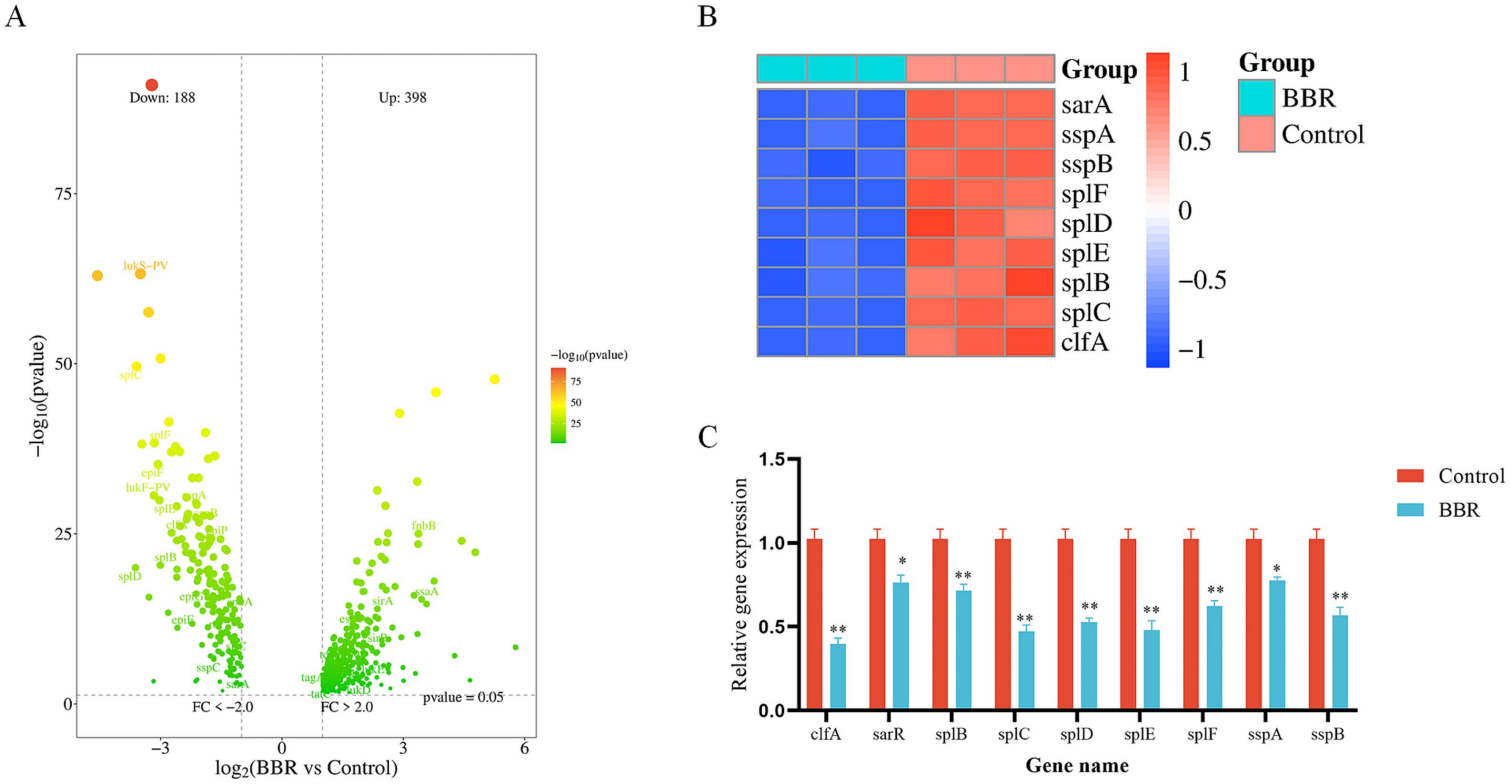
BBR down-regulates the expression of the USA300 virulence gene. **(A)** Volcano plot showing the differential gene expression after BBR treatment; **(B)** Heat map of the virulence gene after BBR treatment; **(C)** qRT-PCR was used to verify the effect of BBR on virulence genes. ^**^p < 0.01, *p < 0.05.

USA300 is a hypervirulent MRSA clone commonly associated with community-acquired infections. The *agr* system in USA300 plays a pivotal role in toxin expression [e.g., *α*-hemolysin (Hla)] and promotes tissue invasion (Figure 2A) (Rossato et al., 2020). The inhibitory effect of BBR on *agr* mRNA levels is therefore likely central to its anti-virulence activity. We next assessed the effect of BBR on the expression of the agr quorum sensing system and its downstream effector, *α*-Hla, at the mRNA level. As shown in Figure 2B, exposure to 64 μg/mL BBR resulted in a modest reduction in the mRNA levels of all three genes examined, with relative expression of *agrA*, *RNAIII*, and *α-Hla* decreasing to 0.81, 0.82 and 0.82-fold of the control, respectively. In contrast, treatment with 128 μg/mL BBR revealed a marked differential sensitivity among these genes. While *α-Hla* transcript levels were only moderately affected (0.73-fold of control), the core agr components *agrA* and *RNAIII* were nearly completely suppressed, with residual expression plummeting to just 0.06-fold and 0.11-fold, respectively. These results suggest that BBR inhibits the expression of *agrA* in a dose-dependent manner at the mRNA level. To assess the effect of BBR on biofilm formation, we performed crystal violet staining and quantified the biomass by measuring the optical density at OD543 nm. As shown in Figure 3C, BBR treatment induced a dose-dependent reduction in biofilm biomass. Relative to the positive control, increasing concentrations of BBR resulted in progressively lower OD values: 0.35 at 16 μg/mL, 0.28 at 32 μg/mL, 0.21 at 64 μg/mL, and 0.06 at 128 μg/mL. At the highest concentration tested (128 μg/mL), the biofilm mass was reduced to a level comparable to that of the negative control, indicating near-complete inhibition of biofilm formation. These results demonstrate that BBR effectively inhibits *S. aureus* biofilm formation in a concentration-dependent manner. To assess the effect of BBR on biofilm formation, we performed crystal violet staining and quantified the biomass by measuring the optical density at OD543 nm. As shown in Figure 3C, BBR treatment induced a dose-dependent reduction in biofilm biomass. Relative to the positive control, increasing concentrations of BBR resulted in progressively lower OD values: 0.35 at 16 μg/mL, 0.28 at 32 μg/mL, 0.21 at 64 μg/mL, and 0.06 at 128 μg/mL. At the highest concentration tested (128 μg/mL), the biofilm mass was reduced to a level comparable to that of the negative control, indicating near-complete inhibition of biofilm formation. These results demonstrate that BBR effectively inhibits *S. aureus* biofilm formation in a concentration-dependent manner.

### BBR down-regulates the expression of the USA300 virulence gene

RNA-seq analysis revealed that treatment with BBR resulted in 398 upregulated and 188 downregulated genes in the USA300 strain (Figure 3A). Importantly, multiple virulence-associated genes were significantly downregulated (*p* < 0.05). These genes are primarily involved in pathogen adhesion, proteolysis—which contributes to immune evasion and tissue destruction—and virulence regulation. This supports the conclusion that BBR reduces pathogenicity by inhibiting virulence gene expression. Among these genes, *clfA* was downregulated by approximately ninefold, suggesting that BBR markedly impairs the adhesion capacity of *S. aureus*, thereby limiting colonization in host tissues and reducing infection risk. In addition, proteases encoded by *splB–splF*, *sspA*, and *sspB*—core virulence determinants of *S. aureus*—were also suppressed. These proteases facilitate bacterial immune evasion and dissemination by degrading host immune molecules (e.g., antibodies and complement) and disrupting structural components of host tissues (e.g., collagen and elastin) (Supplementary Table S2; Figure 3B). Subsequent qRT-PCR analyses further validated these findings, confirming that BBR significantly downregulated the expression of multiple virulence genes, with *clfA* showing the most pronounced reduction (Figure 3C).

### Molecular docking results of BBR with target proteins

**Figure 4:**
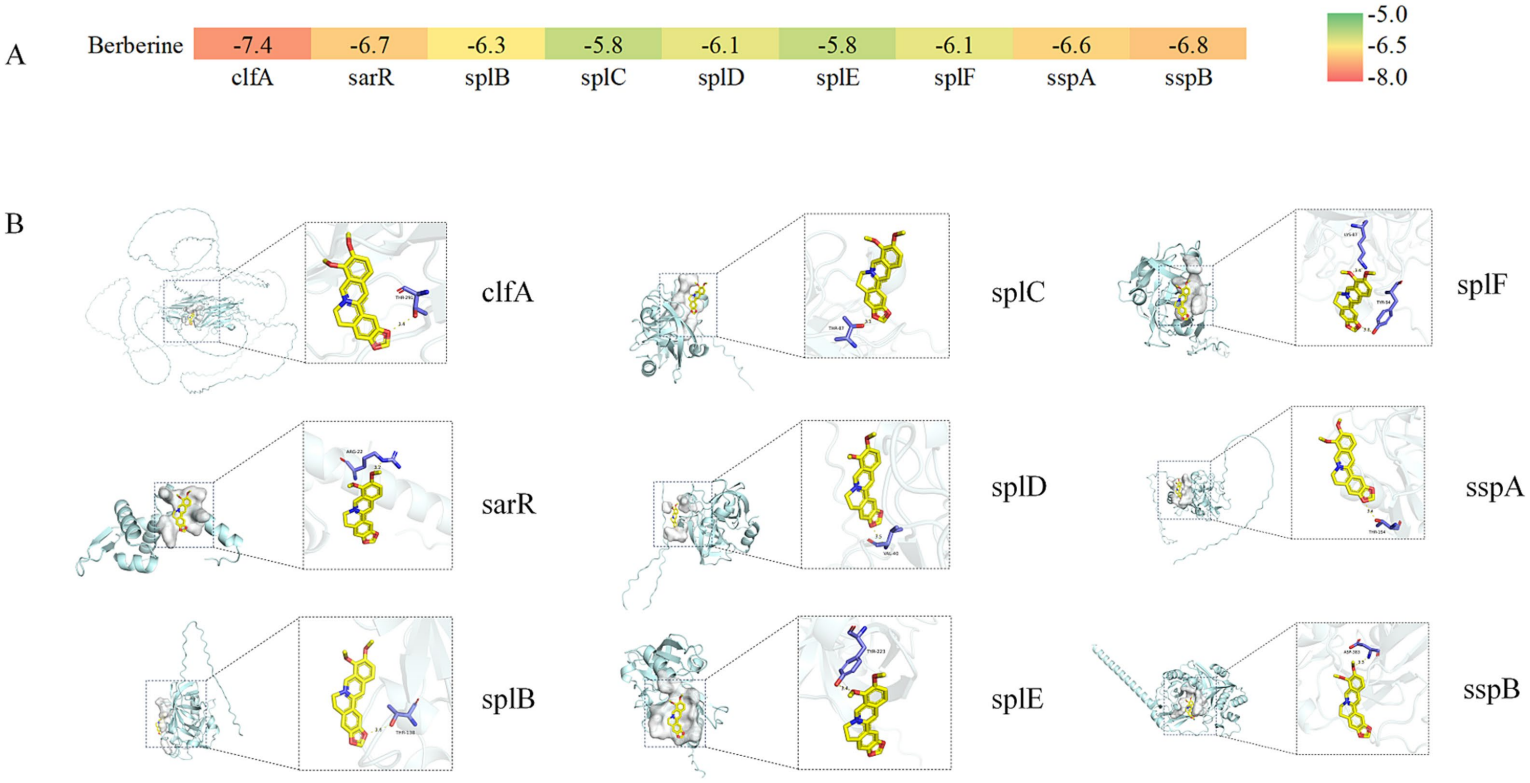
The role of berberine in key enzymes. **(A)** Binding energy thermogram of key enzymes with BBR; **(B)** 3D molecular docking simulation of key enzymes with BBR.

It is well established that lower binding affinity values indicate greater conformational stability between ligands and receptors. Molecular docking analysis demonstrated that BBR exhibits strong binding interactions with several key virulence-related proteins, with all binding affinities being negative and below −5 kcal/mol (Figure 4A). The five proteins showing the strongest binding capacity to BBR were ClfA, SarR, SspB, SspA, and SplB. Structural visualization further revealed that BBR interacts with target protein residues through multiple interactions (Figure 4B). Specifically, BBR forms hydrogen bonds with THR-291 of ClfA, ARG-22 of SarR, THR-138 of SplB, THR-87 of SplC, VAL-40 of SplD, TYR-223 of SplE, LYS-87 and TYR-94 of SplF, and ASP-383 of SspB.

### Effect of BBR on microbial amyloid fibril formation

**Figure 5:**
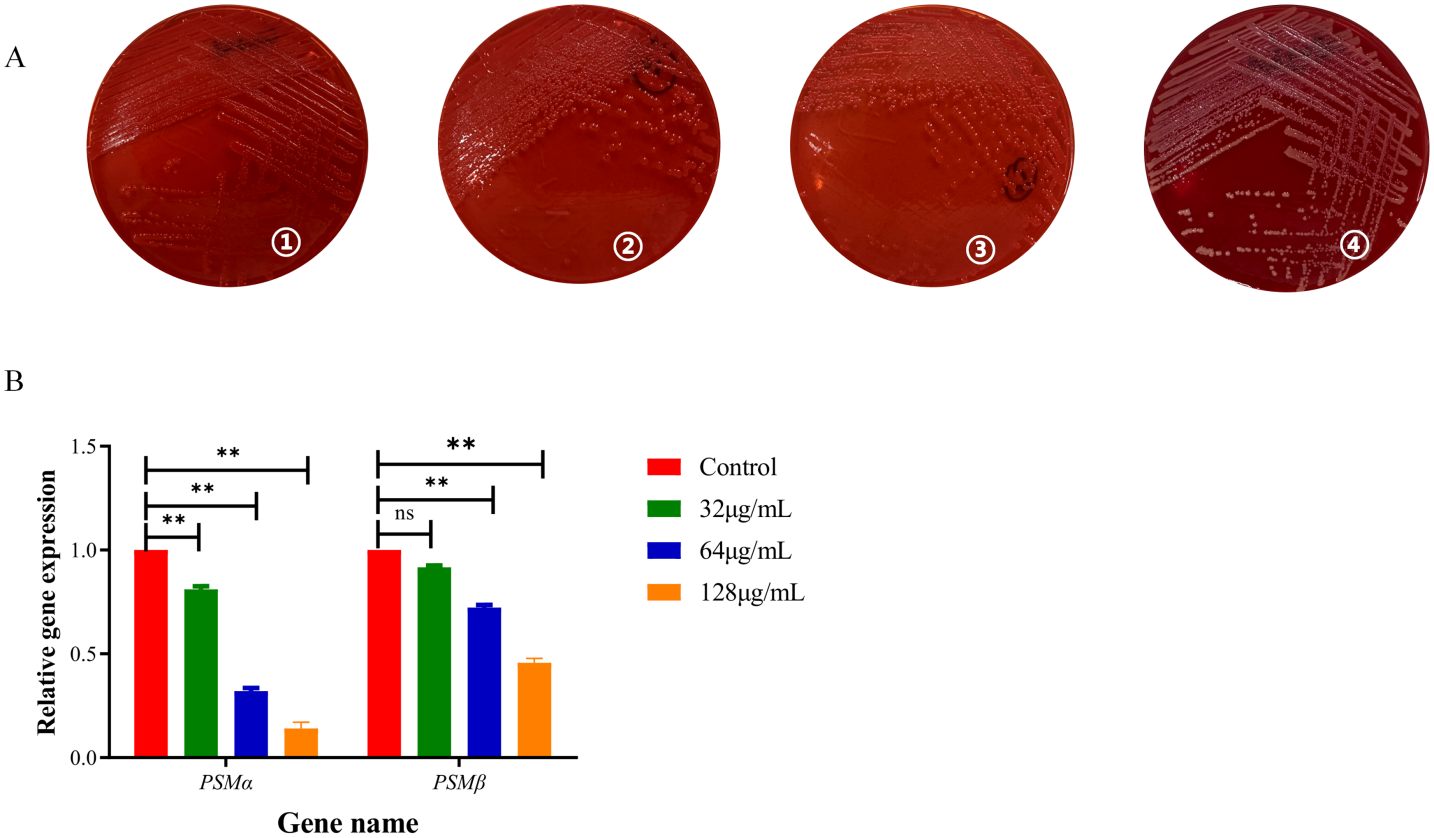
BBR inhibits the production of phenol-soluble modulins. **(A)** Congo red staining of USA300 amyloid fibril: (1) control; (2) 32 μg/mL; (3) 64 μg/mL; (4) 128 μg/mL; **(B)** qRT-PCR was used to verify the effect of BBR on PSM. **p < 0.01, *p < 0.05.

Congo red staining is widely recognized as a sensitive diagnostic tool for detecting microbially generated amyloids (Chu et al., 2016). When cultured in BBR-free medium, MRSA exhibited a classic red, dry, and rough colony phenotype on Congo red-supplemented agar, indicative of normal amyloid fibril formation. In contrast, MRSA grown in medium containing 32 μg/mL BBR produced smooth, pink colonies with markedly reduced amyloid fibril production. A further decrease in fibril formation was observed at 64 μg/mL BBR. Strikingly, when the BBR concentration exceeded 128 μg/mL, MRSA colonies lacking amyloid fibrils were observed (Figure 5A). PSM amyloid fibrils are known to disrupt host cell membrane integrity, thereby exacerbating infection-related tissue damage. Consistent with the phenotypic findings, qRT-PCR analysis demonstrated that BBR significantly inhibited the expression of *psmα* and *psmβ* (Figure 5B).

### BBR inhibits the formation of USA300 biofilms

**Figure 6:**
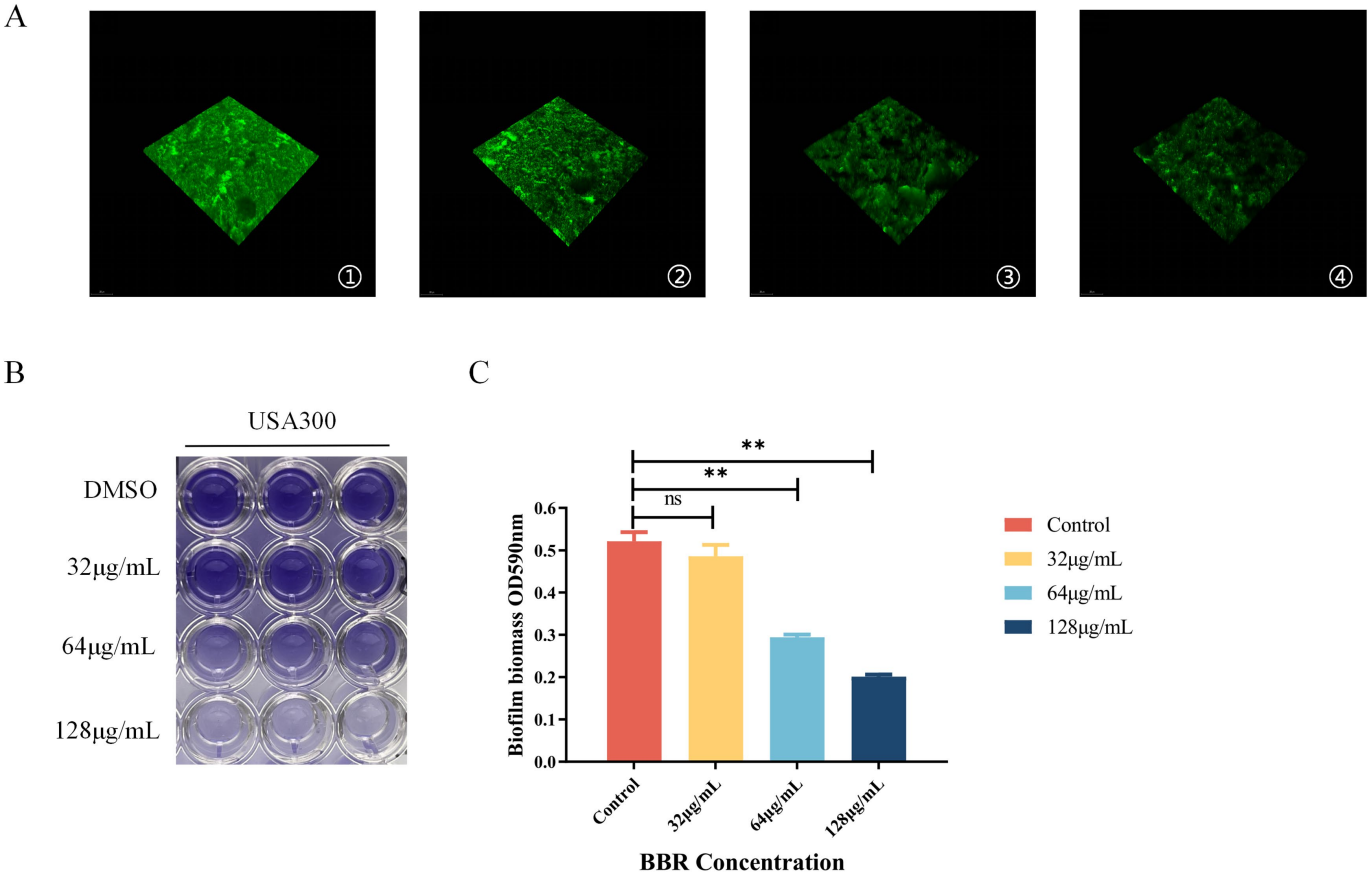
BBR inhibits the formation of USA300 biofilms. **(A)** The effect of BBR on the USA300 biofilm was observed by CLSM: (1) control; (2) 32 μg/mL; (3) 64 μg/mL; (4) 128 μg/mL. **(B,C)** Semi-quantitative experiment of crystal violet staining on biofilms. **p < 0.01, *p < 0.05.

Biofilms play a critical role in protecting MRSA against antimicrobial agents. Biofilm formation was quantified using crystal violet staining, with absorbance measured at 590 nm. The negative control exhibited an OD of 0.521, whereas groups treated with 32, 64, and 128 μg/mL BBR displayed progressively lower OD values of 0.486, 0.2994, 0.201, respectively (Figures 6B,C). To further visualize biofilm formation, acridine orange staining was employed in combination with confocal laser scanning microscopy (CLSM). Acridine orange, a strong fluorescent biofilm biomass indicator, stains both live and dead microbial cells within the biofilm. CLSM imaging revealed that in the absence of BBR, MRSA produced dense microbial colonies covering the entire surface of the coverslips. In contrast, treatment with 32 μg/mL BBR markedly reduced colony numbers, resulting in a scattered distribution. A further reduction was observed at 64 μg/mL, and at concentrations above 128 μg/mL, only sparse colonies were detected on the coverslip surfaces (Figure 6A).

### BBR significantly alleviates MRSA-induced pneumonia in mice

**Figure 7:**
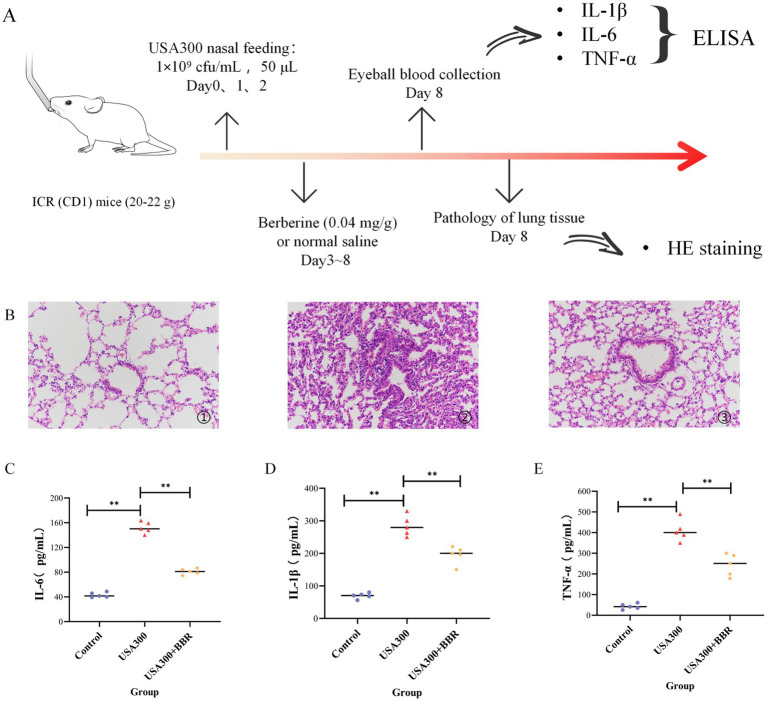
The dose-dependent efficacy of berberine in a pneumonia infection model. **(A)** Schematic diagram of the mouse pneumonia infection model; **(B)** HE staining of pathological sections of mouse lung tissue; **(C-E)** Enzyme-linked immunosorbent assay of mouse serum (IL-6, IL-1β, and TNF-α). **p < 0.01, *p < 0.05.

Our previous research established that BBR exerts significant antibacterial activity against MRSA, with a minimum inhibitory concentration of approximately 128 μg/mL (Zhou et al., 2025). To further evaluate its therapeutic potential, we assessed the effect of BBR in a murine pneumonia model, with a particular focus on its immunomodulatory activity (Figure 7A). Intranasal administration of 1 × 10^9^ CFU/50 μL/mouse of MRSA reliably induced pneumonia, as evidenced by elevated levels of pro-inflammatory cytokines and chemokines, including interleukin-6 (IL-6), tumor necrosis factor-*α* (TNF-α), monocyte chemoattractant protein-1 (MCP-1), and interleukin-1β (IL-1β). Treatment with BBR significantly reduced the expression of these inflammatory mediators (Figure 7C). Histopathological analysis further confirmed the protective effect of BBR. In MRSA-infected mice, alveolar septa were markedly dilated and exhibited severe inflammatory cell infiltration compared with the healthy control group. By contrast, BBR-treated mice displayed substantially reduced pulmonary damage, with alleviated alveolar thickening and diminished inflammatory infiltration (Figure 7B). Collectively, these findings demonstrate that BBR provides significant protection against MRSA-induced pneumonia, at least in part through the suppression of excessive inflammatory responses.

### BBR is non-toxic to L929 cells

**Figure 8:**
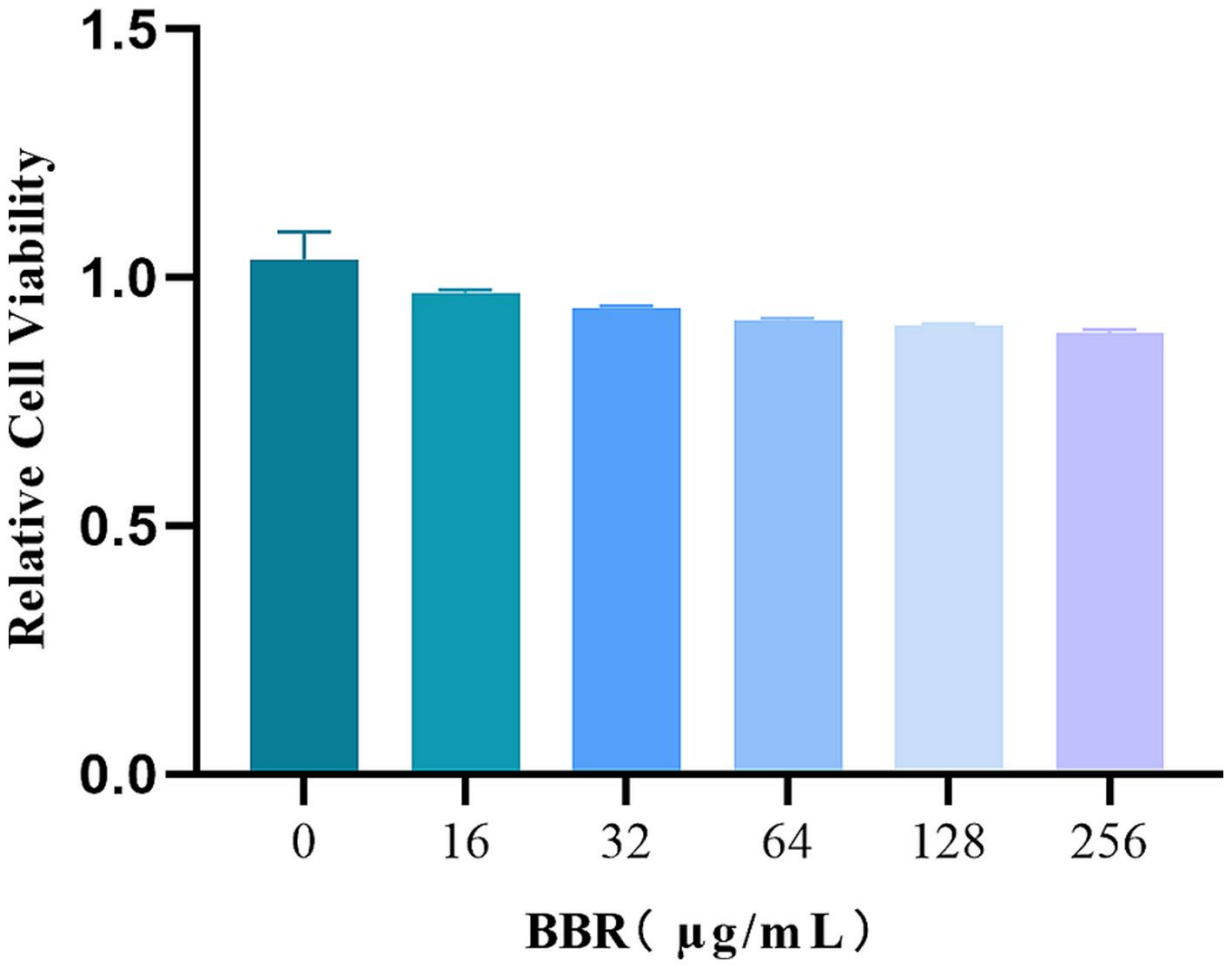
Safety evaluation of BBR. The activity of different amounts of L929 with or without BBR treatment.

To assess cytotoxicity, L929 mouse fibroblasts were exposed to varying concentrations of BBR for 24 h. No significant differences in cell morphology or cell number were observed between the BBR-treated groups and the untreated control. Cell viability was further evaluated using MTT assays, which confirmed the absence of cytotoxic effects after 24 h of BBR exposure. Notably, even at the highest tested concentration (256 μg/mL), BBR did not induce cytotoxicity, with cell viability remaining above 90%. These findings collectively demonstrate the favorable safety profile of BBR (Figure 8).

## Discussion

The emergence and global dissemination of MRSA represent a major public health challenge, as this pathogen is capable of causing severe, difficult-to-treat infections (Zhang et al., 2023). Current MRSA research primarily focuses on two strategies: reducing antimicrobial resistance and attenuating virulence. In this study, we demonstrated that BBR significantly downregulates the expression of key *S. aureus* virulence genes, including *hla*, *psmα*, *psmβ*, *RNAIII*, *clfA*, and *splB–splF*, by targeting the Agr QS system. This inhibition weakens the pathogenic potential of the USA300 strain and contributes to the overall antibacterial activity of BBR.

The regulatory network controlling virulence factor expression in *S. aureus* is highly complex, involving two-component regulatory systems (TCS), QS pathways, the global regulator SarA and its homologues, as well as additional auxiliary regulators (Watanabe et al., 2024). These systems interact intricately to ensure precise control of virulence expression. In *S. aureus*, the QS system is primarily encoded by the *agr* gene cluster, which responds to AIP signals to regulate the mRNA levels of *RNAII* and *RNAIII*. These transcripts, in turn, modulate the expression of multiple virulence factors (Wang and Muir, 2016). This study demonstrates for the first time that BBR exhibits potent inhibitory activity against MRSA. qRT-PCR analysis revealed significant, concentration-dependent down-regulation of *agrA* and *RNAIII* expression, indicating that BBR impairs the function of the Agr quorum sensing system at the mRNA level in a dose-dependent manner.

MRSA expresses a wide range of virulence factors, including adhesins, cytotoxic molecules, and immune-modulating proteins. These virulence determinants vary between strains and are generally classified into categories such as adhesion factors, serine proteases, and hemolysins. In this study, transcriptome sequencing was applied for the first time to assess differential gene expression in MRSA following BBR treatment. The results demonstrated that BBR significantly downregulated virulence-related genes in the USA300 strain, thereby reducing its virulence and attenuating pathogenic potential. These transcriptomic findings were further validated by qRT-PCR analysis, which confirmed BBR’s suppressive effects on the expression of key virulence genes. In parallel, molecular docking simulations provided mechanistic insight by revealing direct interactions between BBR and proteins encoded by these virulence genes. Notably, these interactions were characterized by strong binding affinities, supporting the hypothesis that BBR exerts anti-virulence effects not only at the mRNA level but also at the protein-functional level.

In this study, we demonstrated that BBR significantly reduced the hemolytic activity of USA300 in a concentration-dependent manner. In murine erythrocyte hemolysis assays, culture supernatants from BBR-treated USA300 displayed markedly diminished hemolytic activity, with 128 μg/mL BBR reducing hemolysis to approximately 10% of the untreated control. Consistent with these functional observations, qRT-PCR analysis confirmed that BBR significantly downregulated *hla* mRNA levels in USA300. Notably, the degree of *hla* suppression correlated directly with the reduction in hemolytic activity. Thus, the ability of BBR to inhibit *hla* mRNA levels further supports its role as an effective modulator of the Agr QS pathway. This study further reveals that BBR can effectively inhibit MRSA biofilm formation through multiple mechanisms. Phenotypic assay results showed that following BBR treatment, the biofilm biomass of clinical MRSA isolates was significantly reduced, as confirmed by both crystal violet staining and fluorescence microscopy. Mechanistic investigations indicated that BBR markedly suppresses the expression of *psm* genes and reduces the formation of amyloid fibers on Congo red agar plates—amyloid fibers being a critical component for maintaining the structural integrity and mechanical strength of the biofilm matrix. Therefore, BBR may compromise the biofilm scaffold, rendering bacteria embedded within the film more susceptible to elimination by antibacterial agents or the host immune system.

Subsequent animal experiments further confirmed the protective effect of BBR in a murine model of MRSA-induced pneumonia. BBR treatment significantly ameliorated lung histopathological changes, as evidenced by reduced inflammatory cell infiltration, alleviated alveolar septal thickening, and marked suppression of excessive release of pro-inflammatory cytokines such as IL-6, TNF-α, and IL-1β. Our previous research has established that BBR possesses direct antibacterial activity against MRSA (MIC = 128 μg/mL). However, the protective effects observed in the pneumonia model cannot be solely attributed to this direct antibacterial action. Given that cytokine storm is a key driver of tissue damage in MRSA pneumonia, our findings suggest that the therapeutic efficacy of BBR stems from a dual mechanism of action:on one hand, BBR reduces bacterial load by directly inhibiting MRSA growth;on the other hand, it modulates the host immune response, attenuates excessive inflammation, and thereby prevents inflammation-induced tissue damage. This finding is consistent with numerous reports documenting the anti-inflammatory properties of BBR. From a translational perspective, these results position BBR as a promising anti-virulence and host-directed therapeutic agent for MRSA pneumonia. Unlike conventional antibiotics that exert selective pressure and promote resistance development, BBR targets bacterial pathogenicity rather than directly threatening bacterial survival, offering the potential to reduce the risk of resistance emergence. Furthermore, its ability to modulate host inflammatory responses may provide additional therapeutic value in the management of severe infections where immune-mediated tissue damage is a primary concern. However, several challenges remain before clinical application can be considered. First, the relatively high MIC of BBR against MRSA (128 μg/mL) raises concerns regarding its *in vivo* antibacterial efficacy from a bioavailability standpoint. Although the protective effects observed in our animal studies are partially attributable to immunomodulation, achieving direct bactericidal concentrations at the infection site would likely require optimization of BBR formulation or delivery strategies (e.g., nanocarrier systems or combination with penetration enhancers) to ensure that its pharmacokinetic profile meets therapeutic requirements. Second, the specific molecular targets of BBR in both bacterial and host cells require further elucidation, ideally through unbiased proteomic or chemical biology approaches. Additionally, as this study employed an acute infection model, the efficacy of BBR against chronic or recurrent MRSA infections warrants further investigation; future studies should also assess its potential synergistic effects with conventional antibiotics.

In conclusion, this study not only validates the potential of BBR as an antibacterial agent but also reveals its novel role as an immunomodulator in combating MRSA-induced pneumonia. If the aforementioned translational gaps can be addressed, BBR holds promise as an important component of next-generation therapeutic strategies for drug-resistant *Staphylococcus aureus* infections.

## Data Availability

The data presented in the study are deposited in the NCBI repository, accession number PRJNA1429021 (https://www.ncbi.nlm.nih.gov/bioproject/PRJNA1429021).
